# Prevalence of smoking and its associated factors among students of the University of Dongola, Northern State, Sudan: a cross-sectional study

**DOI:** 10.1097/MS9.0000000000001862

**Published:** 2024-03-18

**Authors:** May M.F. Abdelraouf, Rofida A.M. Abdalla, Douaa M.S. Mohamed, Abubaker K.A. Ahmed, Mohamed A.M. Abuzaid, Mohamed A. Issak, Ibrahim A. Eljack, Elshazaly Saeed, Mohamed O. Abdelaziz

**Affiliations:** a Faculty of Medicine & Health Science; b Department of Medicine, Faculty of Medicine & Health Science, University of Dongola, Northern State; c Federal Ministry of Health, Sudan; d Department of Community Medicine, University of Bisha College of Medicine, Bisha; e Prince Abdullah bin Khaled Coeliac Disease Research Chair, College of Medicine, King Saud University, Riyadh, Saudi Arabia

**Keywords:** associated factors, prevalence, smoking, sudan, university students

## Abstract

**Background:**

Smoking is one of the leading causes of morbidity and mortality worldwide, and its prevalence has increased globally, particularly among university students.

**Objective:**

The study aimed to assess the smoking prevalence and its associated factors among students at University of Dongola, Northern State, Sudan.

**Methods:**

A multi-centred cross-sectional study was conducted among students of University of Dongola. Data was collected via an online Google form questionnaire. Descriptive and comparative analyses were performed using SPSS, version 26. Statistical significance was considered at *p* less than or equal to 0.05.

**Results:**

A total of 642 students participated in the study, of which 51.9% were females. Most of the students (73.6%) were aged 20–25 years and came from health & medical faculties (60.7%). The overall prevalence of smoking was 11.7%. The determined risk factors for smoking included male gender (*P*≤0.001), older age (*P*≤0.001), non-health and non-medical faculties (*P*≤0.001), uneducated fathers (*P*=0.032), and low socio-economic status (*P*=0.001). The most common reason for smoking was stress (36%), with cigarettes being the most commonly used type (88%). Personal savings were the main source of smoking expenses (73.3%). Most smokers (88%) were aware of the harmful effects of smoking.

**Conclusion:**

The overall smoking prevalence was relatively low among students at University of Dongola. Male gender, older age, non-health and non-medical faculties, uneducated fathers, and low socio-economic status were significant risk factors for smoking. The majority of smokers were aware of the harmful effects of smoking.

## Introduction

Highlights

- Tobacco smoking is a significant global health concern, resulting in the deaths of over 8 million people annually.
- In 2020, the prevalence of smoking in the general population was 32.6% among men and 6.5% among women. Vulnerable groups include youths and young adults in universities, with prevalence rates ranging from 10 to 47.5%.
- In Sudan, the general population has a smoking prevalence of 9.6%.
- The prevalence of smoking among students from the University of Dongola, Sudan was 11.7%.
- Risk factors identified for smoking in students from University of Dongola include male sex, older age, non-health and medical faculties, uneducated fathers, low socio-economic status, and stress as a major reason for smoking.

Tobacco smoking is a major global health concern. It is the world’s most frequent cause of preventable early morbidity and mortality, responsible for killing over 8 million people per year^1,2^. It is known to cause various types of cancer, particularly lung cancer, which is considered the primary cause of it^3^. In addition, exposure to secondhand smoke (passive smoking) has been significantly linked to an increased risk of numerous diseases and health issues^4^.

Globally, the prevalence of smoking in the general population in 2020 was 32.6% and 6.5% among men and women, respectively^5^. Although there has been a 27.2% reduction in smoking prevalence, it remains a significant health concern worldwide^5^.

On a regional scale, the smoking prevalence in Africa in 2020 was 18.5%, the lowest compared to other WHO regions^6^. Similarly, the prevalence rate in the Middle East & North Africa (MENA) region was found to be 19.2%^7^.

In Sudan, the general population has a smoking prevalence of 9.6%^8^. Furthermore, a systematic review including 20 articles revealed prevalence rates of tobacco use ranging from 1 to 25% among adolescents and 10–47.5% among adults^9^.

Youths and young adults, which make up the majority of the population in the universities, are mainly the vulnerable group to smoking and are constantly targeted by the tobacco companies^10,11^. A study among Egyptian university students reported a smoking prevalence of 17.4%^12^. Similarly, a systematic review and meta-analysis in Ethiopia revealed a smoking prevalence of 12.55% among university students^13^. Locally, prevalence rates of tobacco use of 13.7% and smoking of 10% were recorded among university students from two studies in Khartoum, Sudan^14,15^.

More than half of college students who smoke do not identify themselves as smokers, describing their smoking habits as a social pastime^16^. The denial perception may be attributed to the fact that most college students intend to quit smoking before graduation, underestimating the difficulties of quitting^17^. Studies have shown that individuals who start smoking at a young age are more likely to have a harder time quitting^18^.

To address the issue of smoking prevalence, various strategies have been recommended by the WHO^19^. These strategies include increasing taxes on tobacco products, implementing bans on advertising and promotion, enforcing restrictions on smoking in public places, educating consumers about the long-term health consequences of smoking, and providing and promoting smoking cessation therapies and clinics.

Given the lack of studies assessing the prevalence of smoking among the University of Dongola students, it is important to evaluate their health status and predictors throughout their academic journeys. This study aims to determine the prevalence of smoking and identify associated risk factors among students at University of Dongola.

## Methods

### Study design

A multi-centred cross-sectional design was conducted between March and October 2022 at University of Dongola. The study was registered with the Philippine Health Research Registry under the registration ID of PHRR231110-006129. In addition, the study adheres to the STROCSS criteria for comprehensive reporting^20^.

### Study setting

The study setting was University of Dongola, Northern State, Sudan, one of the oldest and largest publicly funded universities in Sudan. It was founded in the late 19th century and consists of several faculties. Its campuses are distributed in the different districts of the Northern state in Sudan.

### Study participants

The study population comprised all students registered in all of the faculties of University of Dongola during the study period, which amounted to 11,477. The sample size was calculated using a confidence level of 95%, a marginal error of 5%, and a proportion of 50%. A convenience sampling procedure was utilized, with the calculated sample size of 372 increased to 642 to ensure greater accuracy and validity and to compensate for low response.

### Inclusion and exclusion criteria

The study included any officially registered student at any of the faculties of the University of Dongola during the study period who provided consent to participate in the study. The study excluded graduates and those who did not provide consent. The STROCSS checklist has been adopted on the inclusion and exclusion criteria^20^.

### Data collection

A structured questionnaire was used to collect data from participants, consisting of 16 items, grouped into two sections: social demographic characteristics and smoking status and its characteristics. For this study, any individual who reported to have smoked 100 cigarettes in their lifetime and currently smokes (current smoker) or had quit smoking at the time of data collection (past smoker) or smoked less than 100 cigarettes in their lifetime or never smoked (non-smoker). The questionnaire was translated into Arabic for better understanding and distributed via an online Google form to students through academic groups on WhatsApp and Facebook.

### Data analysis

Data were analyzed using the Statistical Package for Social Science (SPSS), version 26. Statistical tests like χ^2^ test, binary and multinomial regression tests were performed, where the statistical association was considered significant at the *p* value level of less than 0.05.

### Ethical approval

Since this study involved human participants, ethical approval was inevitable. For this reason, the study received ethical approval from the Research Ethical Committee of the X State Ministry of Health (Approval No. 10/2022) and the administration of University of Dongola, and informed consent was obtained from each participant before completing the questionnaire.

## Results

A total of 642 students from different faculties of the University of Dongola participated in the study. Half (51.9%) of them were female students. The majority (89.1%) were aged younger than 25 years. Almost two-thirds (60.7%) were from Health and Medical faculties. More than half (57.3%) were off-campus residents, including those living with their families and in private accommodations. The fathers of most participants were alive (87.5%) and educated (96.8%). The income level of the majority (82.8%) was average. Other sociodemographic data can be found in Table 1.

**Table 1:** Socio-demographics of the participant students from University of Dongola (*n*=642), 2023

Variable	Frequency, *N* (%)
Total	642 (100)
Sex	
Male	309 (48.1)
Female	333 (51.9)
Age:	
<25 years	572 (89.1)
>25 years	70 (10.9)
Type of faculty	
Health or medical faculties	390 (60.7)
Non-health or non-medical faculties	252 (39.3)
Academic level	
First three year	327 (50.9)
Last three year	315(49.1)
Marital status	
Single	599 (93.3)
Married	43 (6.7)
Residence during University Period	
Off-Campus private	110 (17.1)
Off-campus with family	258(40.2)
Public hostel	276(42.7)
Type of residence of origin	
Rural	233 (36.3)
Urban	409 (63.7)
Living status of the father	
Alive	562 (87.5)
Died	80 (12.5)
Monthly income level of the father: (dead excluded)	
Low	58 (10.3)
Average	466 (82.9)
High	38 (6.8)
Educational level of the father: (dead excluded)	
Uneducated	18 (3.2)
Educated	544 (96.8)

**Figure 1:**
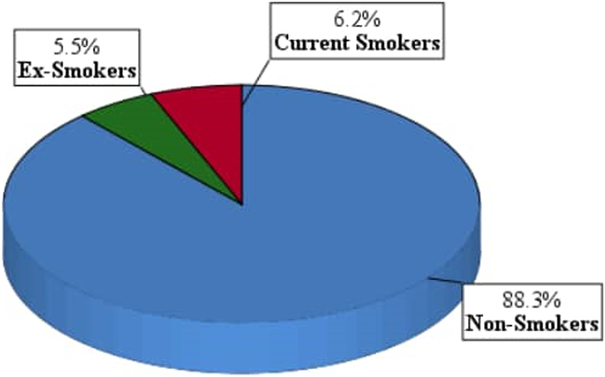
Prevalence of smoking among students of the University of Dongola, 2022

The overall prevalence of smoking among the students of University of Dongola was 11.7%. Ex-smokers’ prevalence was 5.5%, while current smokers were 6.2% (Fig. 1).

Using χ^2^ test, as demonstrated in Table 2, there was a significant association between smoking and gender (*P*≤0.001); age (*P*=0.001); type of faculty (*P*≤0.001); monthly income of the father (*P*= 0.002); and educational level of the father (*P*=0.032). No significant association was found between smoking and academic level (*P*=0.127); marital status (*P*=0.320); type of original residence (*P*=0.281); residence during university (*P*=0.575); and fathers’ living status (*P*=898) (Table 2).

**Table 2:** Relationship between basic characteristics and smoking in the participant students from University of Dongola (*n*=640), 2023

Variable	Smokers, *N* (%)	Non-smokers, *N* (%)	Total, *N* (%)	*P*
Total	75	567	642	
Sex				
Male	72 (23.3)	237 (76.7)	309 (48.1)	< 0.001^a^
Female	3 (0.9)	330 (99.1)	333 (51.9)	
Age				
<25 years	58 (10.1)	514 (89.9)	572 (15.6)	0.001
>25 years	17 (24.3)	53 (75.7)	70 (10.9)	
Type of faculty				
Health or medical faculties	25 (6.4)	365 (93.6)	390 (60.7)	< 0.001
Non-health or non-medical faculties	50 (19.8)	202 (80.2)	252 (39.3)	
Academic level				
First 3 years	32 (9.8)	295 (90.2)	327 (13.2)	0.127
Last 3 years	43 (13.7)	272 (86.3)	315 (19.6)	
Marital status				
Single	72 (12)	527 (88)	599 (93.3)	0.461^a^
Married	3 (7)	40 (93)	43 (6.7)	
Residence during University period				
Off-campus private	15 (13.6)	95 (86.4)	110 (17.1)	0.575
Off-campus with family	32 (12.4)	226 (87.6)	258 (40.2)	
Public hostel	28 (10.2)	246 (89.8)	276 (42.7)	
Type of residence of origin				
Rural	23 (9.9)	210 (90.1)	233 (36.3)	0.281
Urban	52 (12.7)	357 (87.3)	409 (63.7)	
Living status of the father				
Alive	66 (11.7)	496 (88.3)	562 (87.5)	0. 898
Died	9 (11.2)	71 (88.8)	80 (12.5)	
Monthly income level of the father: (dead excluded)				
Low	15 (25.9)	43 (74.1)	58 (10.3)	0.002
Average	46 (9.9)	420 (90.1)	466 (82.9)	
High	5 (13.2)	33 (86.8)	38 (6.8)	
Educational level of the father: (dead excluded)				
Uneducated	5 (27.8)	13 (72.2)	18 (3.2)	0.032
Educated	61 (11.2)	483 (88.8)	544 (96.8)	

a Fisher’s exact test was used to calculate the *p* value since the observed cell counts are less than 5.

Table 3 shows the assessment of risk factors of smoking in the participants using Binary and multinomial logistic regression tests. Based on sex, smoking was 33 times higher in males than in females [odds ratio (OR)=33.42; 95% CI: 10.40–107.34; *P*≤0.001]; It was also higher in the age-group older than 25 years (OR=0.35; 95% CI: 0.191–0.647; *P*=0.001). According to the faculty type, non-health and non-medical students were found to smoke three times more frequently than their medical or health science peers (OR=3.61; 95% CI: 2.17–6.01; *P*≤0.001). (Table 3).

**Table 3:** Assessment of risk factors of smoking in the students from University of Dongola, using logistic regression tests (*n*=642), 2023

Variable	OR (95% CI)	*P*
Sex		
Female	Reference	< 0.001
Male	33.42 (10.40–107.34)	
Age		
<25 years	Reference	0.001
>25 years	0.35 (0.191–0.647)	
Type of faculty		
Health or medical faculties	Reference	< 0.001
Non-health or non-medical faculties	3.61 (2.17–6.02)	
Monthly income level of the father		
Low	0.31 (0.16–0.61)	0.001
Average	Reference	
High	0.72 (0.27–1.94)	0.520
Educational level of the father		
Uneducated	3.05 (1.05–8.84)	
Educated	Reference	0.032

OR, odds ratio.

Finally, smoking prevalence was three times more common in students whose fathers had no education (OR=3.05; 95% CI: 1.05–8.84; *P*=0.032). Similarly, it was also more common in students whose fathers had low income than those whose fathers had average or higher income (OR=0.31; 95% CI: 0.16–0.61; *P*=0.001).

Among the smokers, 88% smoked cigarettes, 10.7% smoked Shisha, and 1.3% smoked other types. The frequency of smoking per day was once for 10.7%, twice for 22.7%, and three times or more for 66.7%. The sources of smoking allowances were friends (4%), family (22.7%) and personal savings (73.7%). The reasons for smoking were curiosity for 9.3% of the students, stress for 36%, influence by friends for 72.7%, boredom for 28%, and other reasons for 24%. The majority of smokers (88%) were aware of the harmfulness of smoking. (Table 4).

**Table 4:** Characteristics of smoking participant students from University of Dongola (*n*=640), 2023

Variable	Frequency	N (%)
Type of smoke		
Cigarette	66	88
Shesha	8	10.7
Others	1	1.3
Frequency of smoking per day		
Once	8	10.7
Twice	17	22.7
Thrice or more	50	66.7
Source of smoking allowances		
Friends	3	4
Family	17	22.7
Savings	55	73.3
Reasons for smoking		
Curiosity	7	9.3
Stress	27	36
Influenced by friends	2	2.7
Boredom	21	28
Other reasons	18	24
Awareness of the harmfulness of smoking		
Yes	66	88
No	9	12

## Discussion

This study aimed to investigate the smoking prevalence and risk factors among students of University of Dongola, Northern State, Sudan. The study found an overall smoking prevalence of 11.7%. These findings are consistent with previous studies; one conducted on medical students at National Ribat University, Khartoum, and another in Ethiopia, a neighbouring country^13,15^. However, the prevalence rate is significantly lower than those reported in China and Turkey^21,22^. The observed disparity in prevalence rates may be attributed to the higher smoking rates observed among Southeast Asians and Europeans compared to Africans^6^.

Significant gender differences were observed in smoking prevalence, with male students exhibiting a significantly higher prevalence compared to female students. Similar findings have been reported in a study conducted in Minia, Egypt, and a systematic review from Saudi Arabia^23,24^. Conversely, studies from Slovakia, Bosnia, and Portugal showed no significant association between sex and smoking^25–27^. The higher prevalence of smoking among male students in our study may be attributed to sociocultural and religious factors specific to Sudan. In Sudan, smoking is discouraged and even considered socially stigmatizing for females.

Furthermore, the study revealed a significant association between smoking and age, indicating that older students were more likely to smoke. This finding aligns with a study conducted in Yemen, which demonstrated a significant increase in smoking rates with age^28^. In contrast, a study from Malaysia found no significant association between age and smoking^29^.

Moreover, smoking prevalence was found to be significantly higher among non-health and non-medical faculty students compared to their counterparts in health and medical faculties. This observation is consistent with previous studies conducted in Egypt and Saudi Arabia^12,30^. The difference can be explained by the fact that students in health and medical faculties possess better understanding and are more oriented to the negative health effects associated with smoking. However, a study conducted in Malaysia, where smoking is more prevalent, reported no significant relationship between faculty type and smoking^29^.

The study also explored the relationship between smoking prevalence and the father’s education level. Students with uneducated fathers exhibited a significantly higher smoking prevalence compared to those with educated fathers (*P*=0.008). Conversely, two previous studies from Cairo, Egypt, and Turkey reported no significant association between the father’s education level and smoking^22,31^.

Furthermore, the current study observed a higher smoking prevalence among students from low socio-economic backgrounds (*P*=0.001). In contrast, a study from Balikesir University in Turkey significantly associated smoking with high socio-economic levels^22^.

Among the smokers in the current study, the majority (66.7%) reported smoking more than three times a day. This is consistent with findings from a study conducted in Malaysia, where the majority of smokers (29.6%) reported smoking more than once per day^29^. Cigarettes were the most common type of smoking product among smokers in the current study (88%), followed by Shesha (10.7%) and other types (1.3%). This preference for cigarettes may be attributed to the easy availability and affordability of cigarettes compared to other types of smoking products.

Stress was the most frequently reported reason for smoking among participants in the current study (36%). This finding supports previous research conducted in Malaysia, where stress was also identified as the primary reason for smoking^29^. However, it is worth noting that a local study among the general population reported curiosity as the most common reason for substance abuse overall, with stress ranking as the third most common reason^14^.

Regarding the efforts to tackle the tobacco smoking issues, Sudan became a member of the WHO Framework Convention on Tobacco Control in January 2006. The country has implemented several measures to address the issue. These include creating smoke-free environments by restricting tobacco use in indoor workplaces, public places, and certain forms of public transport; the prohibition of advertising and promoting tobacco products in any form and its sale in or near educational, healthcare and religious facilities; and expanding health warning coverage from 30 percent to 75 percent on tobacco products packaging^32^.

### Strengths and limitations

Some of the main strengths of this study are its design, its instrument, and the sample size that represents the targeted population.

Regarding the limitations, this study was based on the self-reporting system of students, which could face the risk of memory bias. There is also a risk of the students providing socially desirable answers, especially female students, which could limit the study. In addition, the period limitation of the study could also hinder the generalization of the findings all the time.

## Conclusion

In the current study, the overall prevalence of smoking among students of University of Dongola was shown to be 11.7%. The determined risk factors for smoking were male sex, older age, non-health and non-medical faculties, uneducated fathers, and low socio-economic status. The most commonly reported reason for smoking was stress (36%). The majority smoked cigarettes (88%) and at a frequency of more than three times a day (66.7%).

### Recommendations

Based on the results, the researchers recommend the university administrations and state ministry of health should provide awareness campaigns, workshops, and events. In addition, the researchers recommend that the university administration apply strict anti-smoking policies on the university campus, develop smoking cessation clinics, and provide psychotherapists to help students manage stress. Finally, the researchers urge more detailed studies addressing the smoking population to be conducted on the topic.

## Ethical approval

The study received ethical approval from the Research Ethical Committee of the Northern State Ministry of Health, Sudan (Approval No. 10/2022), and a verbal approval from the university administration.

## Consent

Written informed consent was obtained from the patient for publication of this case report and accompanying images. A copy of the written consent is available for review by the Editor-in-Chief of this journal on request.

## Sources of funding

None.

## Author contribution

All authors contributed equally to all the research work.

## Conflicts of interest disclosure

There are no conflicts of interest.

## Research registration unique identifying number (UIN)

Registry: The Philippine Health Research Registry (PHRR) Registry ID: PHRR231110-006129 Hyperlink: https://registry.healthresearch.ph/index.php/registry?view=research&layout=details&cid=6129.

## Guarantor

All authors accept the full responsibility for the work, have the data and possess the decision to publish.

## Data availability

Data presented in this study are available anytime upon request from the corresponding author.

## Provenance and peer review

The authors declare that this study is original, has never been invited, not published before, and is not currently being considered for publication elsewhere. It is not commissioned, externally peer-reviewed.
